# Molecular Characterization of Transgenic Events Using Next Generation Sequencing Approach

**DOI:** 10.1371/journal.pone.0149515

**Published:** 2016-02-23

**Authors:** Satish K. Guttikonda, Pradeep Marri, Jafar Mammadov, Liang Ye, Khaing Soe, Kimberly Richey, James Cruse, Meibao Zhuang, Zhifang Gao, Clive Evans, Steve Rounsley, Siva P. Kumpatla

**Affiliations:** Dow AgroSciences LLC, 9330 Zionsville Road, Indianapolis, Indiana, 46268, United States of America; Jawaharlal Nehru University, INDIA

## Abstract

Demand for the commercial use of genetically modified (GM) crops has been increasing in light of the projected growth of world population to nine billion by 2050. A prerequisite of paramount importance for regulatory submissions is the rigorous safety assessment of GM crops. One of the components of safety assessment is molecular characterization at DNA level which helps to determine the copy number, integrity and stability of a transgene; characterize the integration site within a host genome; and confirm the absence of vector DNA. Historically, molecular characterization has been carried out using Southern blot analysis coupled with Sanger sequencing. While this is a robust approach to characterize the transgenic crops, it is both time- and resource-consuming. The emergence of next-generation sequencing (NGS) technologies has provided highly sensitive and cost- and labor-effective alternative for molecular characterization compared to traditional Southern blot analysis. Herein, we have demonstrated the successful application of both whole genome sequencing and target capture sequencing approaches for the characterization of single and stacked transgenic events and compared the results and inferences with traditional method with respect to key criteria required for regulatory submissions.

## Introduction

Commercialization of transgenic crops can be achieved only after regulatory approval which requires rigorous assessment of their safety [1, 2]. Molecular characterization of transgenic events is an important analysis towards this goal and is conducted at two stages: first, for the selection of desirable events and later for the characterization of selected lead event(s) to support regulatory submissions. A thorough molecular characterization of the transgene locus, determining its sequence, integrity and its location in the genome, is a critical step in the safety assessment process. This characterization also addresses mandatory analysis that determines whether the transgene expression cassette is inserted into the host genome as a single copy, is intact across generations, has made any unintended alterations to the host genome due to insertion, and whether it lacks the backbone sequences derived from the plasmid vector used for the transgenesis. Furthermore, using a segregating population, it has to be proven that the inserted transgene behaves as a Mendelian locus.

A key technique that is widely utilized in molecular characterization is Southern blot (SB) analysis [3]. Although SB, along with polymerase chain reaction (PCR) and Sanger sequencing, is a universally accepted technique for event sorting and molecular characterization studies for regulatory submissions, it is a very time- and labor-intensive and relatively expensive procedure. Moreover, despite being a robust technique and has been successfully used for the molecular characterization of inserted DNA in regulatory studies for many years, SB is not sensitive enough to detect individual nucleotide substitutions and small insertions/deletions that might occur within a transfer DNA (T-DNA) or around a transgene insertion site [4]. Although the disadvantages of SB can be addressed by Sanger sequencing, this sequencing technique does struggle to accurately sequence complex regions of the genomes [5].

**Fig 1:**
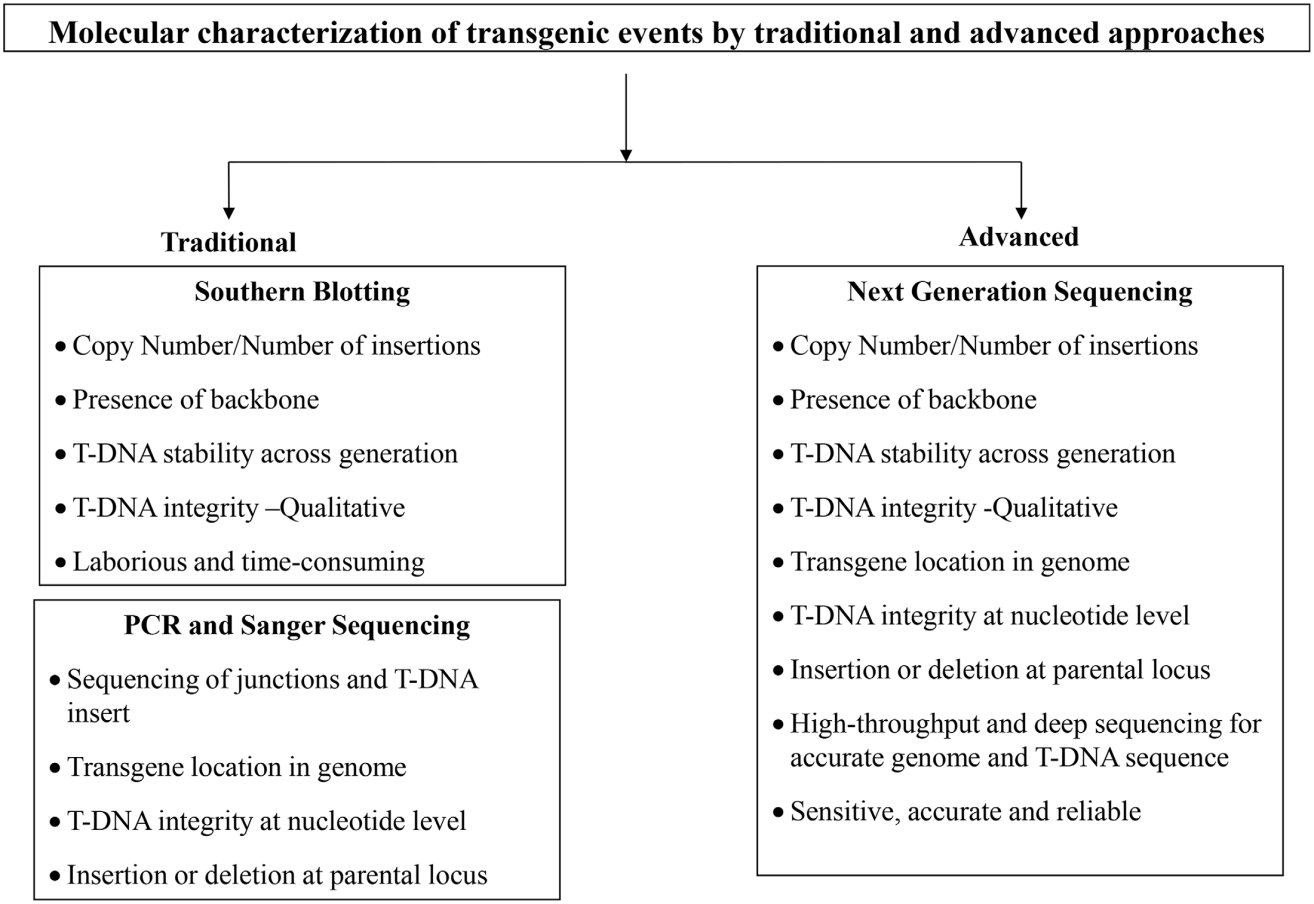
Molecular characterization of transgenic events by traditional and advanced approaches.

The emergence and rapid evolution of next-generation sequencing (NGS) technologies over the past few years have offered novel, rapid, and cost-effective options for molecular characterization of transgenic crops. As NGS has been widely used for the detection of the structural variations [6], this technology can also be applied for molecular characterization of transgenic events. The application of NGS for event characterization has been extensively reported in animal biotechnology. For instance, this technology was successfully applied to characterize transgenic events in cattle and mouse [7–9]. In contrast to conventional PCR and SB methods, NGS has proven to be very sensitive to detect incomplete and multiple integration events [7]. This technology was also used to characterize transgene insertion sites that were located in complex regions of a genome [8, 9]. In plant biotechnology, the number of publications reporting the NGS-based molecular characterization of transgenic events is very limited. Yang et al. [4] confirmed that paired-end re-sequencing was more sensitive than PCR and SB analysis for molecular characterization of transgenic events as it revealed additional unintended insertions in a transgenic rice event. Kovalic et al. [10] successfully demonstrated that transgenic events can be characterized by combining NGS with Sanger sequencing, which consequently can be used as an alternative to the SB method. They applied a whole genome sequencing approach to determine transgene copy number in maize by re-sequencing the junction regions between the transgene and the flanking border genomic sequences. However, Sanger sequencing has been used for assessing the integrity and stability of a T-DNA across generations [10]. Using a combination of high coverage whole genome sequencing and bioinformatics analysis, T-DNA insertion and copy number was previously demonstrated in papaya by the assembly of a draft genome [11]. A novel hybrid NGS x PCR-based method was developed for high-throughput zygosity detection in transgenic maize. However, the application of this method requires a prior information of the exact integration site, adjacent genomic sequences and the transgene copy number [12]. Most recently, targeted sequence capture coupled with NGS was successfully applied for event sorting [13]. Thus, there is sufficient evidence that NGS can be used for event characterization of plant and animal transgenic events. In this paper, we present and compare the results of molecular characterization of two transgenic soybean events, Transgenic Event 1 (TE1) and Transgenic Event 2 (TE2), and their breeding stack (TE1 x TE2) using traditional (SB analyses coupled with Sanger sequencing) and advanced (NGS) methods (Fig 1). In particular, for the first time, we have demonstrated the use of both whole genome sequencing (WGS) and target capture sequencing (TCS) approaches for the characterization of both single and stacked events and compared the results and inferences with traditional method with respect to key criteria required for regulatory submissions.

## Results

### Molecular characterization of two soybean events, TE1 and TE2, and their breeding stack using traditional approach

#### Southern blot analysis

To determine transgene copy number, we performed SB analysis using probes designed to hybridize the inserted DNA (S1 and S2 Figs). The genomic DNA of two single events (TE1 and TE2) was digested with restriction enzymes that cut the inserted DNA to generate distinct patterns and sizes. Although blots were hybridized with different probes covering the entire T-DNA, only the data related to the gene-of-interest (GOI) probes are shown in this paper. For all three generations tested, SB analysis has shown identical patterns and band sizes for all restriction digests on both single events (TE1 and TE2): the probe combinations and hybridizing band patterns indicated that each of these events harbors a single copy of the transgene (Fig A-C in S3 and S4 Figs).

When probes from the plasmid backbone region were used for SB analysis, the expected bands were detected in the respective positive (plasmid) controls whereas no hybridization signal was detected in the transgenic events suggesting the absence of the backbone regions in the transgenic events (Fig A-C in S5 and S6 Figs).

Using the same probe sets and multiple restriction enzymes as used for the analysis of single events, SB analyses were carried out with the soybean breeding stack, TE1 x TE2 (Fig A-C in S7 Fig). A comparison of Southern band patterns from the breeding stack with those of the single TE1 and TE2 events revealed no differences, indicating that the breeding process did not affect the integrity and copy number of T-DNA of the corresponding single events comprising the stack (Fig A-C in S7 Fig).

#### Sanger sequencing

Sanger sequencing has been employed to determine the structural integrity and location of the transgene insert in the genome as well as to identify any rearrangements associated with transgene insertion and any unanticipated changes that may have occurred in the stack compared to single events. The results of Sanger sequencing demonstrated that the T-DNAs within the TE1 and TE2 events were intact at the nucleotide level compared to their counterparts in the plasmids used for transformation (Fig A and B in S8 Fig). Sequence analysis that has compared the regions flanking the insertion site of the transgene with the same regions of the parental genome determined the exact location of the transgene insert in the genome and identified structural variations (insertions) occurred due to the T-DNA insertion in single events (TE1 and TE2) and breeding stack (Fig A-D in S8 Fig). Sequence comparison of the TE1 and TE2 components within the breeding stack TE1 x TE2 with their single event counterparts (TE1 and TE2) demonstrated that the breeding process did not affect the integrity of the T-DNA (Fig C and D in S8 Fig). Taken together, both SB analysis and Sanger sequencing enabled us to successfully characterize both single and stacked events.

### Molecular characterization of the soybean events and their breeding stack using NGS

In parallel to traditional molecular characterization, transgenic single events, TE1 and TE2, and their breeding stack, TE1 x TE2, were also subjected to characterization using Illumina paired end (PE) sequencing—both whole genome sequencing (WGS) as well as targeted capture sequencing (TCS). PE sequencing generates read pairs from both ends of a sheared DNA fragment, which when mapped to the combination of plant reference genome and plasmid sequence can answer several key questions pertaining to transgenic event characterization such as the location of the insert, copy number, transgene integrity, stability and the lack of vector backbone.

#### Location of the insert and copy number

To determine the number of copies of the transgene inserted in the genome and their locations, genomic DNA of the transgenic plant was randomly sheared and sequenced. Such a random shearing produces a mixture of three types of fragments—those derived solely from the plant genome, those derived solely from the transgene and those derived from regions spanning the transgene integration site and thus consisting of both the transgene and the host DNA. When mapped back to the reference genome and transgene sequences, the PE reads generated from this third type of fragment will have one read of a pair mapped to the transgene and its mate mapped to the plant genome. This class of PE reads will subsequently be referred to as ‘junction pairs’. In a subset of these junction pairs, one read of the pair will span the junction with a portion of read derived from the transgene and the other derived from the genome. These will be referred to as ‘junction reads’. The combination of junction pairs and junction reads helps to identify the transgene integration site(s) in the genome. In our WGS experiments, the number of junction pairs varied from event to event. For instance, in the TE1 event, we obtained an average of 72 junction pairs (average of F2 and T3 generations) that had one read mapped within the T-DNA and its mate mapped to the plant genome. Out of these, seven junction reads spanned the 5’ junction region and five reads spanned the 3’ junction region (Table 1). All 5’ and 3’ junction reads got mapped to a single genomic location which suggested a single transgene integration site for the TE1. If there were multiple insertions of T-DNA within a host genome, WGS would have yielded heterogeneous population of junction reads pointing to multiple locations in the genome.

**Table 1:** Insertion location, integration site in soybean genome and 5’ and 3’ Flanking Junction reads by whole genome sequencing of TE1 and TE2 single and TE1 x TE2 breeding stack events.

Trait	Breeding Generation and Sample Number	Integration site in soybean genome	Total Reads	Reads mapped to genome (Coverage)	Reads mapped to Transgene (Coverage)	Number of Junction Pair End (PE) pairs	5’ Junction Reads coverage	3’ Junction Reads coverage
TE 1	F2-1	Gm06:18,552,249..18,556,606	98,725,411	93,762,230 (10X)*	863 (9X)**	72	6****	5
TE 1	T3-3	Gm06:18,552,249..18,556,606	119,386,991	110,949,911 (11X)	938 (9X)	72	8	6
TE 2	F2-10	Gm02:10,027,285..10,027,342	108,314,430	102,156,087 (10X)	517 (4X)***	29	1	2
TE 2	T3-1	Gm02:10,027,285..10,027,342	95,660,998	91,367,370 (9X)	952 (8X)	47	4	5
TE 1 in TE 1 x TE2 stack	Single generation	Gm06:18,552,249..18,556,606	146,746,063	140,544,330 (14X)	1,128 (11X)	38	8	3
TE 2 in TE 1 x TE 2 stack	Single generation	Gm02:10,027,285..10,027,342	146,746,063	140,544,330 (14X)	1,338 (11X)	13	6	5

* The coverage of the genome was calculated by the formula [(number of reads)*length of a read]/genome size] with an average read length and the size of the soybean genome being equal to 100 bp and 975,000,000 bp, respectively.

**The coverage of the T-DNA was calculated by the formula [(number of reads)*length of a read]/length of T-DNA] with the insert size of ~10,000 bp in Trait 1 and ~12,500 bp in Trait 2.

***The coverage of the T-DNA in TE2 in F2 generation is half of that in T3 generation as the sample (F2-10) representing the former is in hemizygous state.

**** The coverage within the junctions was represented by number of reads spanning those regions.

**Fig 2:**
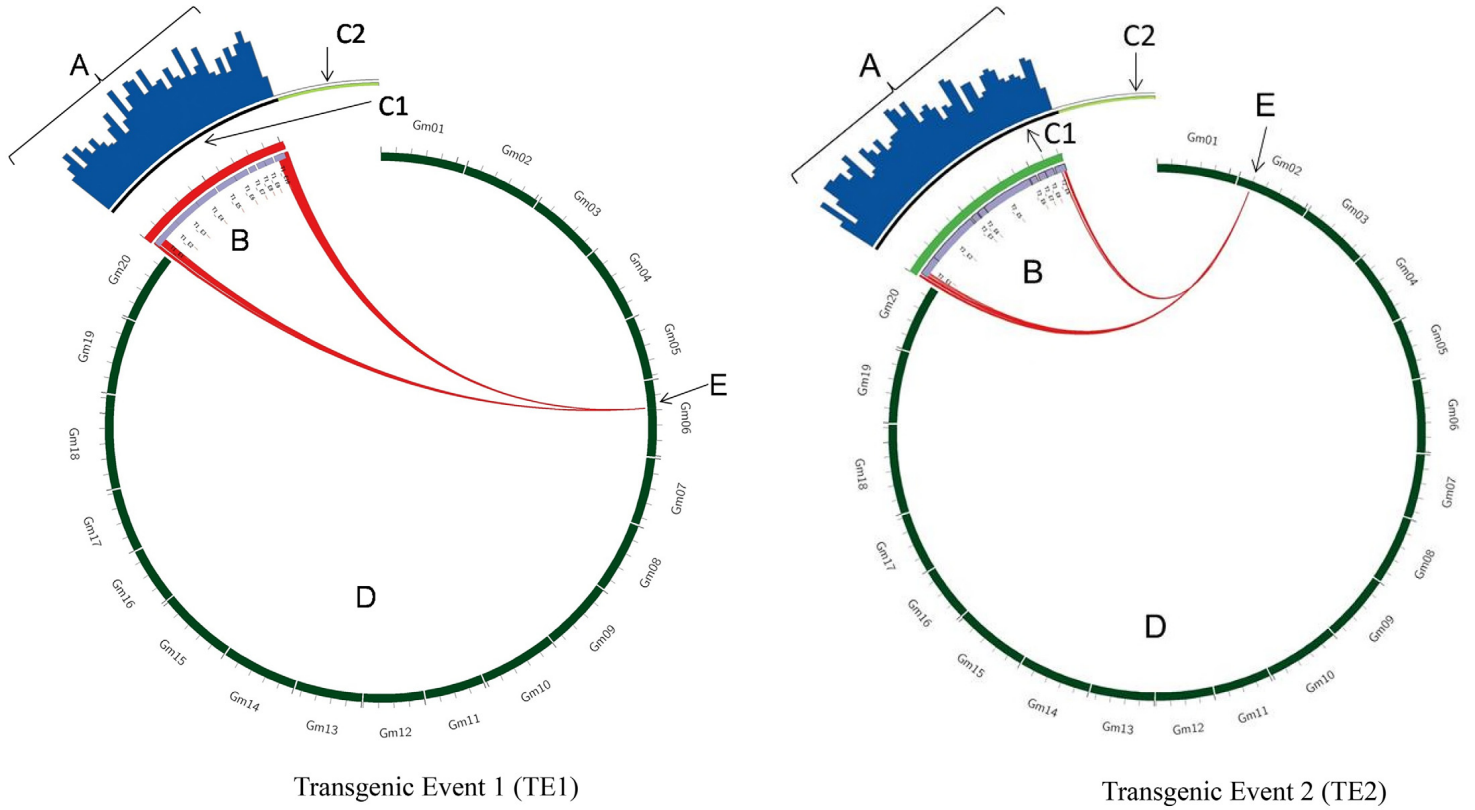
Molecular characterization of transgenic soybean Transgenic Event 1 (TE1) and Transgenic Event 2 (TE2) using whole genome sequencing. Genomic DNA of TE1 and TE2 was randomly sheared and sequenced using Illumina's HiSeq2000 instrument. The genome coverage was ~ 10X, i.e. 10 copies of soybean haploid genome. Short HiSeq2000 reads (A) spanning entire T-DNA within TE1 and TE2 (B) were mapped back to transformation plasmid that contained intended T-DNA (C1) and backbone (C2). Uninterrupted blue bars aligned to the intended T-DNA (C1) of the transformation plasmid confirms the integrity of T-DNA within events. No blue bars over plasmid backbone (C2) confirms the absence of those sequences within the genome of TE1 and TE2 events. Twenty chromosomes (*Gm*1-20) of soybean reference genome (Williams 82 version X) are represented in circular fashion (D). Reads spanning junction regions were mapped back to soybean reference genome, which showed single insertion site on chromosome 6 (E) in TE1 and chromosome 2 (E) in TE2.

The copy number was estimated by comparing the genome coverage of the reference genome to the transgene coverage. For TE1, the reference coverage and transgene coverage were 10x and 9x, respectively, suggesting a single transgene copy. In case of multiple copy insertions, the coverage of a transgene would have exceeded the coverage of a genome, with the increase in fold change reflecting an increased copy number. In both F2 and T3 generations, junction reads got mapped to a single locus of the soybean chromosome 6 (Tables 1 and 2 and Fig 2E) and identified the integration site of the T-DNA very precisely.

**Table 2:** Insertion location, integration site in soybean genome and 5’ and 3’ Flanking Junction reads by Target genome sequencing of TE1 and TE2 single and TE1 x TE2 breeding stack events.

Trait	Breeding Generation and Sample Number	Integration site in soybean genome	Reads mapped to transgene (Avg. coverage)	Number of Junction Pair End (PE) pairs	5’ Junction Reads coverage	3’ Junction Reads coverage
TE 1	F2-1	Gm06:18,552,249..18,556,606	279797 (6053x)	50970	4905	7013
TE 1	T3-3	Gm06:18,552,249..18,556,606	267750 (5792x)	49607	4692	6919
TE 2	F2-10	Gm02:10,027,285..10,027,342	161770 (2821x)	17722	1847	4029
TE 2	T3-1	Gm02:10,027,285..10,027,342	265192 (4625x)	28051	3001	6006
TE 1 in TE 1 x TE2 stack	Single generation	Gm06:18,552,249..18,556,606	362253 (7837x)	62534	9206	10587
TE 2 in TE 1 x TE 2 stack	Single generation	Gm02:10,027,285..10,027,342	455842 (7950x)	25019	6987	18792

With respect to TE2, one of the samples tested (‘F2-10’) was a hemizygous plant and this was reflected in the genome coverage data. The hemizygous sample (F2-10) had half the genome coverage (~4x) compared to both the reference genome and the homozygous sample (T3-1) which had 9x coverage (Table 1). Despite relatively lower coverage, in both F2 and T3 generations, junction reads were mapped to a single locus on chromosome 2 (Table 1 and Fig 2E) confirming a single locus integration of TE2.

**Fig 3:**
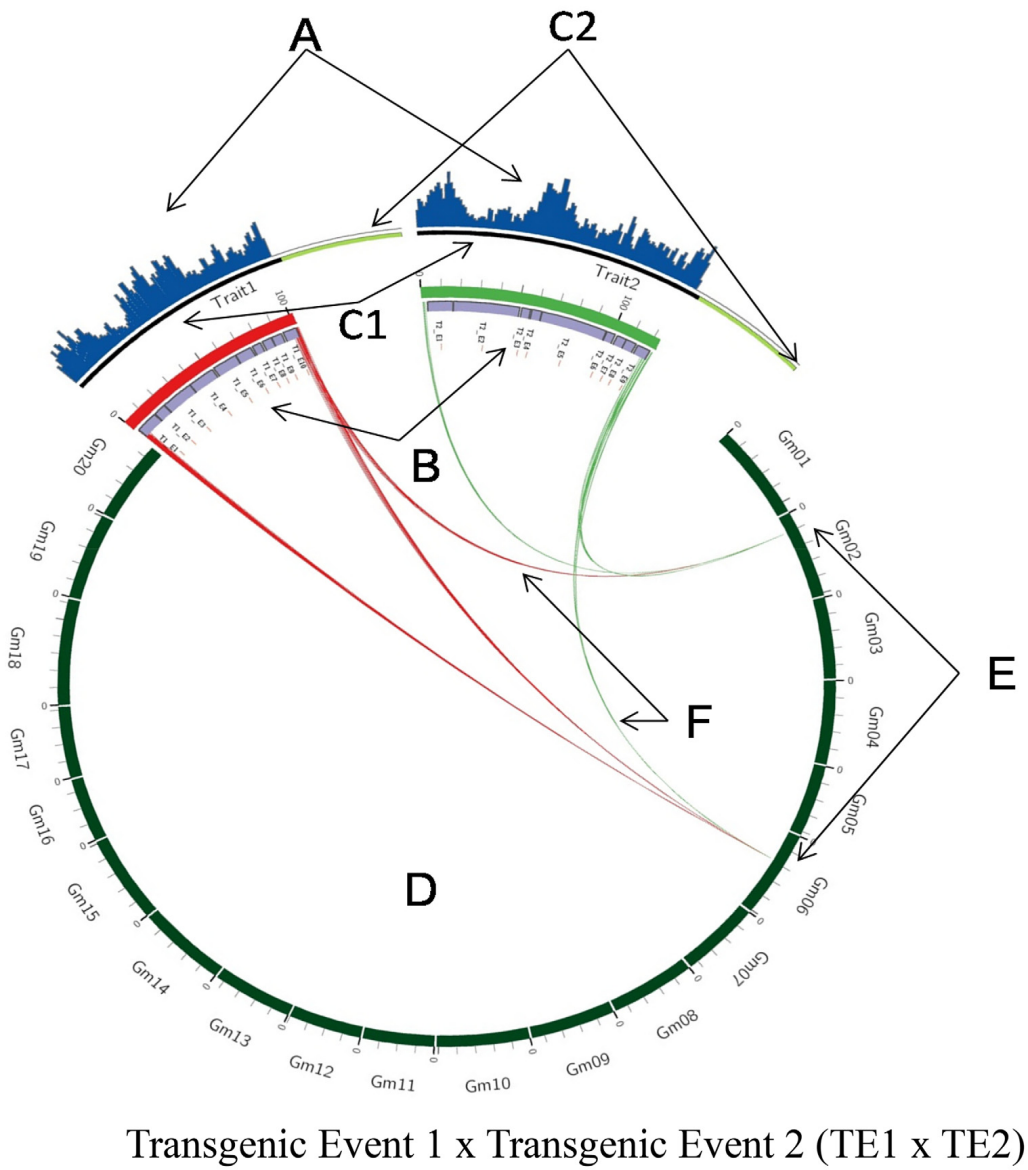
Molecular characterization of soybean breeding stack Transgenic Event 1 x Transgenic Event 2 (TE1 x TE2) using whole genome sequencing. Genomic DNA of TE1 x TE2 was randomly sheared and sequenced using Illumina's HiSeq2000 instrument. The genome coverage was ~ 14X, i.e. 14 copies of soybean haploid genome. Short HiSeq2000 reads (A) spanning entire T-DNA within TE1 and TE2 (B) were mapped back to transformation plasmid that contained intended T-DNA (C1) and backbone (C2). Uninterrupted blue bars aligned to the intended T-DNA (C1) of the transformation plasmid confirms the integrity of T-DNA within TE1 and TE2. No blue bars over plasmid backbone (C2) confirms the absence of those sequences within the genome of TE1 and TE2. Twenty chromosomes (*Gm*1-20) of soybean reference genome (Williams 82 version X) are represented in circular fashion (D). Reads spanning junction regions were mapped back to soybean reference genome, which showed single insertion site on chromosome 6 (E) in TE1 and on chromosome 2 (E) in TE2. T-DNA insert in both TE1 and TE2 share the same fragment at the 3'border region (F).

A similar analysis of the TE1 x TE2 breeding stack also confirmed a single copy (14x genome coverage vs. 11x T-DNA coverage, Table 1), single integration of each of TE1 and TE2 with an average of five junction reads supporting the 5’ and 3’ junctions (Table 1 and Fig 3). However, we did observe that the junction pairs from the 3’ end of TE1 mapped to the genome integration sites of both TE1 and TE2 and a similar pattern of mapping to two integration sites was also observed for the junction pairs from the 3’ end of TE2 (Fig 3F). As both TE1 and TE2 share a common terminator in the 3’ region (T1_E10 = = T2_E9; Fig 3F), the reads obtained from this part of the transgene resulted in non-specific mapping of some TE1 pairs to the TE2 location and vice versa. Results from the sequencing data concurred with the corresponding Southern blot results and confirmed the single copy single integration of the TE1 X TE2 stack. These results suggest that breeding process did not change the copy number and insertion site of T-DNA of the single events.

Although the results obtained through WGS related to the copy number and insertion site characterization are consistent with SB analysis and Sanger sequencing, WGS generates relatively lower coverage of junction reads, which are crucial in defining the copy number of T-DNA. Further increase in sequencing coverage will increase the coverage of junction reads, but will also increase the cost of the experiments. To address this issue, we explored an alternative sequencing approach, namely target capture sequencing (TCS) that was expected to increase the coverage within T-DNA and junction regions without generating a large amount of host genomic sequences. TCS is an approach that uses a WGS library of fragments as described above and a collection of bait probes designed against a desired target sequence. The baits are used as hybridization probes to capture and thus, increase the relative abundance of fragments from the targeted region. The results of TCS, summarized in the Table 2, demonstrated a remarkable increase (several thousand folds) in coverage at junction regions (Table 2). Importantly, TCS data confirmed the single copy status of T-DNA within both single and stacked events. As the sequence capture method focuses on capturing only the transgene sequence and a small portion of the genomic DNA at the integration site, the single copy nature of the transgene was determined by the homogeneity of junction reads and not by the comparison of coverage across transgene and genome as was done for the WGS since it lacked the broad coverage across the whole genome. However, if the copy number of T-DNA was to be defined by the comparison of the coverages of host genome and transgene, any single copy housekeeping gene could be used in the sequence capture and could serve as a control for copy number estimations. The TSC results agree with the WGS results and confirm the single copy, single insertion in TE1, TE2 and their breeding stack TE1 x TE2, and produced significantly higher number of junction reads.

#### Transgene integrity

The sequence coverage and PE mapping information was used to assess the integrity of the T-DNA. The WGS datasets contained an average coverage of 9x and 8x across the T-DNA for TE1 and TE2 events, respectively, and 11x coverage for the breeding stack (Table 1). The sequence coverage indicated the presence of all the T-DNA elements and the sequence of the T-DNA from the events exactly matched the reference sequence suggesting the absence of any variations in the T-DNA. The TCS datasets had significantly higher coverage (>2800x) than the WGS datasets (Tables 3 and 4) and were in agreement with the results from the WGS data.

**Table 3:** Molecular characterization of soybean single events, TE 1 and TE2, and their breeding stack TE1× TE2 using whole genome sequencing sequencing approaches. Each element of the T-DNA is represented by the “X” amount of coverage depth. Both T-DNA inserts within TE1 and TE2 events share several identical elements, such as T1_E5 = T2_E1 (promoters), T1_E7 = T2_E3 = T2_E6 (terminators), T1_E8 = T2_E4 = T2_E7 (promoters), T1_E9 = T2_E8 (gene of interest, GOI), and T1_E10 = T2_E9 (terminators).

TE1			TE1 in TE1 × TE2
Generations	F2_1	T3_3	Single generation
Elements(Donor Organism)	Coverage (nX)		
T1_E1-expression enhancer*(Nicotiana tabacum)*	8	8	14
T1_E2 –terminator(*Arabidopsis thaliana*)	8	9	12
T1_E3 –GOI(*Zea mays*)	6	7	5
T1_E4 –promoter(*Arabidopsis thaliana*)	8	8	15
T1_E5 –promoter(*Arabidopsis thaliana*)	8	8	15
T1_E6 –GOI(*Delftia acidovorans*)	8	7	6
T1_E7 –terminator(*Agrobacterium tumefaciens*)	9	7	7
T1_E8 –promoter(*Cassava Vein Mosaic virus*)	7	9	8
T1_E9 –GOI(*Streptomyces viridochromogenes*)	7	10	7
T1_E10 –terminator(*Agrobacterium tumefaciens*)	8	12	9
TE2			TE2 in TE1 × TE2
Generations	F2_10	T3-1	Single Generation
Elements(Donor Organism)	Coverage (nX)		
T2_E1 –promoter(*Arabidopsis thaliana*)	4	8	14
T2_E2 –GOI(*Bacillus thuringiensis* subsp. aizawai)	4	6	6
T2_E3 –terminator(*Agrobacterium tumefaciens*)	4	8	20
T2_E4 –promoter(*Cassava Vein Mosaic virus*)	5	8	21
T2_E5 –GOI(*Bacillus thuringiensis* subsp. kurstaki)	3	7	9
T2_E6 –terminator(*Agrobacterium tumefaciens*)	4	9	9
T2_E7 –promoter(*Cassava Vein Mosaic virus*)	3	10	8
T2_E8 –GOI(*Streptomyces viridochromogenes*)	2	6	8
T2_E9 –terminator(*Agrobacterium tumefaciens*)	3	5	11

**Table 4:** Molecular characterization of soybean single events, TE 1 and TE2, and their breeding stack TE1× TE2 using target capture sequencing approaches. Each element of the T-DNA is represented by the “X” amount of coverage depth. Both T-DNA inserts within TE1 and TE2 events share several identical elements, such as T1_E5 = T2_E1 (promoters), T1_E7 = T2_E3 = T2_E6 (terminators), T1_E8 = T2_E4 = T2_E7 (promoters), T1_E9 = T2_E8 (gene of interest, GOI), and T1_E10 = T2_E9 (terminators).

TE1			TE1 in TE1 × TE2
Generations	F2_1	T3_3	Single generation
Elements	Coverage (nX)		
T1_E1-expression enhancer	14874	15877	14871
T1_E2 –terminator	7433	7244	6711
T1_E3 –GOI	189	131	78
T1_E4 –promoter	2126	1743	1653
T1_E5 –promoter	2392	1883	1774
T1_E6 –GOI	5171	4476	4242
T1_E7 –terminator	12914	11770	11373
T1_E8 –promoter	15291	14219	13808
T1_E9 –GOI	12804	11951	11788
T1_E10—terminator	11033	9007	8800
TE2			TE2 in TE1 × TE2
Generations	F2_10	T3-1	Single Generation
Elements	Coverage (nX)		
T2_E1 –promoter	1881	3049	4869
T2_E2 –GOI	2409	3738	5401
T2_E3 –terminator	5294	9086	13035
T2_E4 –promoter	5907	10313	14303
T2_E5 –GOI	1426	2358	3653
T2_E6 –terminator	5182	8776	12930
T2_E7 –promoter	6799	11757	17953
T2_E8 –GOI	5905	9692	16089
T2_E9—terminator	3984	6342	13483

**Fig 4:**
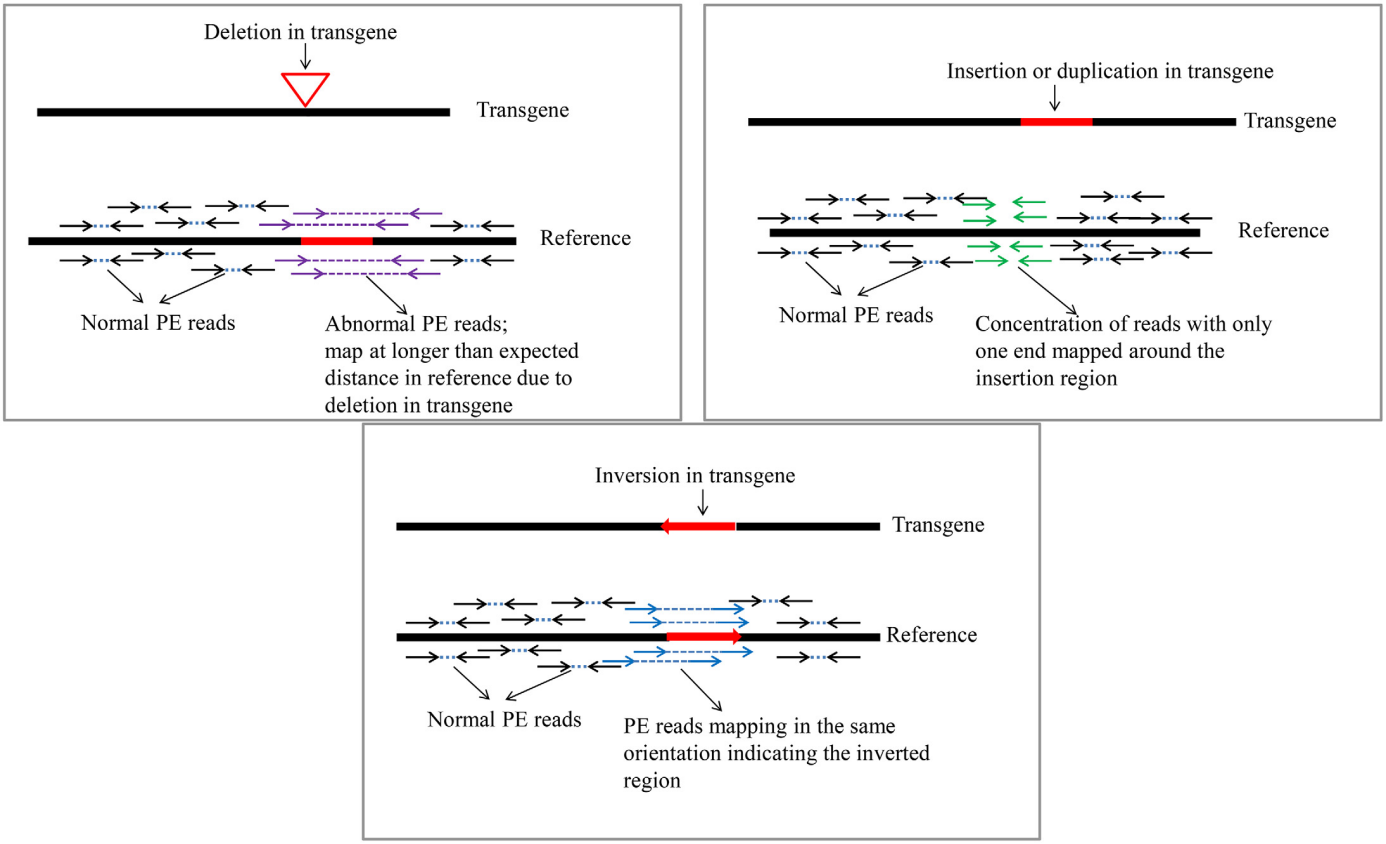
Sensitivity of paired end chemistry of NGS in detecting small DNA aberrations (insertion, deletion, and inversion) within T-DNA.

Analysis of relative spacing and orientation of mapped PE reads can indicate evidence of re-arrangements. In Fig 4, we show how PE reads would be mapped in three distinct types of re-arrangements. No such anomalous pairs were observed during the sequencing of the three events indicating the absence of any rearrangements within the T-DNA confirming the integrity of the T-DNA. WGS and TCS data were in concordance with SB and Sanger sequencing and confirmed the integrity of T-DNA inserts within single and stack events at the nucleotide level.

TSC experiments gave us much higher coverage than WGS, which in turn gives us much higher confidence that the T-DNA inserts in all three events were intact. Compared to Sanger sequencing, where the DNA fragment is sequenced from both sides and, on an average, achieves 2x coverage at the overlapping and 1x at non-overlapping portions, NGS (especially TCS) provides much more robust data to determine the integrity of the T-DNA at the nucleotide level. TCS generates a higher level of coverage for a given cost because it sequences only a captured fragment of DNA.

#### Presence/absence of vector backbone

Sequencing data was also used to determine whether any portion of the vector backbone is present in the transgenic plant. As WGS generates sequences from the entire genome, any vector backbone segments that are present in the transgenic plant will be sequenced and these reads will map back to the plasmid reference sequence. In this study, we did not see any reads mapping to the vector backbone of the construct suggesting a clean integration of the T-DNA and absence of any vector backbone in the transgenic plant. These results were consistent for TE1 and TE2 across all generations and their breeding stack and were further confirmed by TSC experiments also (Figs 2C2 and 3C2).

#### Stability

Using both WGS and TCS approaches we generated the same information from TE1 and TE2 single event samples representing two generations, F2 and T3 (Tables 1–4). No differences in copy number and integrity of T-DNA were observed in both generations suggesting that T-DNA is stably inherited across generations. These results correspond with the results of the SB analysis.

## Discussion

The results reported here highlight the application of NGS to molecular characterization of transgenic events. We show that NGS offers an effective, robust, and sensitive method to identify the transgene insert location, copy number, integrity, and stability.

There are several advantages of NGS over traditional SB + Sanger sequencing analysis. One of them is the sensitivity aspect of technology. Due to the nature of chemistry, particularly PE sequencing, technology allows to detect small DNA re-arrangements (insertions/deletions and inversions) within T-DNA. Although no re-arrangements within T-DNA were found in this study, in the Fig 4 we demonstrated several possible scenarios when PE sequencing could detect small DNA aberrations. Additionally, high level of coverage makes the base calling more reliable and robust. One can rightly argue that Sanger sequencing is also capable of detecting those mutations. However, this can be true only if the complexity of the event allows to generate PCR amplicons covering T-DNA, junction regions and flanking borders to be further Sanger-sequenced. In case when transgene lands in the highly repetitive regions of a genome, PCR amplification of the region spanning borders and junctions becomes very cumbersome task. This is also true for the events that were created by particle bombardment as the latter can generate deletions and scramble inserted and chromosomal DNA [14]. In the above-mentioned circumstances, deep sequencing offers much higher sensitivity and ability to resolve the complex issues. Superior strategies exists using NGS technologies compared to PCR based methods for resolving problems that are caused by integration of transgene in repetitive region of genomic DNA. Recently emerged single-molecule based NGS technologies generate longer reads (2,000–5,000 bp) at increased coverage depth. The latter is particularly important in resolving the challenges in analysis of the repetitive and low complexity regions of a genome [15 16]. Another area where NGS was reported to be more advantageous is the cost of experiments and the amount of labor spent for molecular characterization. Particularly, Kovalic et al [10] reported that conducting the molecular characterization using NGS approaches reduces the cost and labor by 50% compared to Southern blot-based approach. DNA sample preparation for NGS technologies requires shearing of the genomic DNA, using starting material in much lower quantities than needed for SB (up to more than 10-fold less), the subsequent selection of sheared DNA-fragments with appropriate size and the library construction of DNA-fragments for sequencing can be performed with commercially available kits in very high-throughput manner. Although we did not do the direct per-sample cost comparison, we also observed that NGS-based molecular characterization was time-and labor-effective. However, we need to state that in our experiments, SB analysis was conducted under Good Laboratory Practice (GLP) quality management system while Sanger and NGS were not. Any experiments that are done under GLP or ISO quality management systems involve substantial amount of time spent for quality assurance processes which significantly slow down the process. Cost savings could be also relative and depend on the nature of the event. In case of “difficult” events, NGS might require higher coverage and more time spent for data processing that would dramatically increase the cost of the experiments.

In our WGS experiments, we achieved slightly lower coverage within junction regions (~7x) than across the transgene (9x). At this point, literature lacks sufficient information related to a “gold standard” for the level of coverage within junction regions to make a solid determination of transgene copy number. Recently Kovalic et al [10] reported ~70x coverage within junction regions. While it is possible to expand the coverage of the genome in WGS experiments, this increases the cost of molecular characterization for regulatory submissions. Also the application of WGS for molecular characterization of transgenic crops may be less affordable for companies or institutions with modest budgets due to the high cost of experiments, resources needed to conduct extensive bioinformatics data processing, and purchase and maintenance of storage space for enormous amount of sequencing data [17]. Recently, Zastrow-Hayes et al [13] demonstrated the use of TCS method coupled with NGS technology for high throughput event sorting during trait development process. In this study, we have demonstrated that TCS technology can answer all key questions pertaining to molecular characterization of transgenic crops posed by regulatory agencies. In comparison to WGS TCS could achieve very high coverage within junction regions which boosted our confidence in characterizing the insertion site and defining a copy number of a transgene. As TCS focuses on the target region (e.g. T-DNA) only, the technology generates much less sequencing data and, consequently, requires less storage space and resources to complete bioinformatics analysis. Defining a “gold standard” for the level of coverage within junction regions will ultimately depend on the nature, and specifically the complexity of junction regions. Junction regions with complex DNA re-arrangements occurring during transformation might require much higher coverage to increase the confidence level in decision making. On the contrary, “clean” junction regions might not need that high level of coverage to define the copy number. For high quality data analysis and assembly, sufficiently high coverage is required, which can increase the cost of sequencing. Therefore, the depth of coverage should be set on case-by-case basis and a balance between the requirement, cost and coverage should be made [18]. NGS-based molecular characterization of transgenic events is a promising new trend in regulatory sciences. Thus, for regulators, it is crucially important to understand the similarities between NGS-based and SB-based molecular characterization of transgenic events which will be very helpful during the review of regulatory dossiers. In Table 5, we tried to draw parallels between TCS-based, WGS-based and SB-based molecular characterization. The process comparison clearly demonstrates that the principle of the TCS method closely mirrors the probe versus genomic DNA hybridization principle of SB—analysis (Table 5).

**Table 5:** Comparison of concepts between Southern blot analysis, Target capture sequencing and Whole Genome Sequencing.

Step	Southern Blot analysis	Target Capture Sequencing	Whole Genome Sequencing
1	~2500 bp overlapping probes are designed to cover entire length of T-DNA.	~120 bp capture probes (bates) are designed to cover entire T-DNA	No analogy
2	Genomic DNA of an event is digested by restriction enzymes	Genomic DNA of an event is randomly sheared and library prepared	Genomic DNA of an event is randomly sheared and library prepared
3	Genomic DNA transfer and immobilization on the matrix	No analogy	No analogy
4	Hybridization of a labeled probe with genomic DNA immobilized on the matrix	Hybridization of biotinylated probes with sheared genomic DNA. Add magnetic streptavidin beads to bind to biotinylated probe	No analogy
6	Wash away unhybridized probes and non-specific hybridizations	Capture target with magnet and wash away off-target sequences	No analogy
7	DNA band detection and visualization	Sequence captured DNA fragments	Sequencing of entire genome
8	Outcome: a band	Outcome: DNA sequence	Outcome: DNA sequence

Although we have demonstrated here that reads generated by both WGS and TCS technologies can be successfully employed for characterizing the entire T-DNA, the short reads may pose some challenges to resolve junction regions within the complex repeat-rich regions of the genome or with repetitive regions within the T-DNA [19]. However, the combination of paired-end sequencing with larger read lengths and insert sizes and the advancements in sequencing platforms that enable longer read lengths can mitigate these disadvantages [20].

## Conclusion

Molecular characterization of transgenic events using NGS technology, namely whole genome sequencing and targeted capture sequencing, can successfully answer all major regulatory questions related to transgene copy number, T-DNA integrity, stability of T-DNA insert across different generations, and the presence/absence of plasmid backbone sequence. Unlike SB analysis, where the decision on the status of the transgene is made based on banding pattern, the outcome of NGS-based molecular characterization is an actual sequence which is confirmed at several fold coverage. In terms of coverage TCS looks more attractive compared to WGS as it is capable of providing ultra-high coverage within T-DNA and junction regions for a reasonable cost. Due to the paired-end chemistry both WGS and TCS possess much higher sensitivity in detecting small DNA re-arrangements within T-DNA and junction regions rather Southern Blot analysis. Although fairly short read lengths could cause a problem in resolving complex junction regions or with insertions within repetitive sequences, this could be overcome by increasing the coverage within the troublesome region using TCS approach. Overall, NGS based molecular characterization is a robust and reliable approach and with further chemistry improvement, in particular an increase in the length of reads, it can easily replace labor- and time-consuming Southern blot analysis in molecular characterization of transgenic crops for regulatory submissions.

## Methods

### Plant materials

Soybean transgenic single events, TE1 and TE2, were generated by *Agrobacterium* transformation. Soybean breeding stack, TE1 x TE2, was developed by conventional breeding of TE1 and TE2. Non-transgenic soybean control plants are the conventional soybean varieties with a genetic background of the single and stacked events. For Southern blot analysis of TE1 and TE2 single events, four homozygous generations and one segregating generation with three replications for each generation were grown in greenhouse conditions. For Southern blot analysis of TE1 x TE2 stack single homozygous line with three replications was used. For Next-generation sequencing analysis, one homozygous line and one heterozygous line with one replication for each of TE1 and TE2 single events and one homozygous line for TE1 x TE2 stack was used.

### Genomic DNA extraction

Genomic DNA from frozen soybean leaf tissue from single events, breeding stack event, non-transgenic control plants were extracted following modified CTAB method [21]. Following extraction, the DNA was quantified spectrofluorometrically using PicoGreen reagent (Invitrogen). The DNA was then visualized on an agarose gel to check for genomic DNA quality. Genomic DNA was used for Southern blot, whole genome sequencing and target capture sequencing analysis.

### Southern blot analysis

Ten micrograms of genomic DNA from transgenic single and stacked events, non-transgenic control, non-transgenic control spiked with plasmid were digested with required restriction enzymes. Multiple restriction enzymes were selected to determine the copy number, integrity of inserted T-DNA as well as the absence of transformation plasmid backbone in transgenic events. For the single transgenic events (TE1 and TE2) probes specific to TE1 and TE2 events and their backbone regions were labeled with DIG-dUTP using a PCR DIG Probe Synthesis Kit (Roche Diagnostics, Indianapolis, IN). Southern blot analysis was performed essentially as described by Memelink et. al [22]. Hybridization and detection were completed according to the manufacturer’s instructions (Roche Diagnostics, Indianapolis, IN). For the stacked event, probes were radioactively labeled with [α-32P]dCTP using the Prime-It RmT Random Primer Labeling Kit (Agilent Technologies, Santa Clara, CA) and purified using ProbeQuant G-50 Micro Columns (GE Healthcare, Chalfont St Giles, Buckinghamshire). Hybridization was conducted with Perfect Hyb Plus hybridization and the membranes were then exposed to X-ray film sandwiched between two intensifying screens for one to three days in -80°C freezer. The film was then developed with an All-pro imaging film developer (ALLPRO Imaging, Melville, NY).

### Sanger sequencing

Sanger sequencing was applied to determine the intactness of T-DNA at nucleotide level and characterize the insertion site in parental locus. The entire length of the T-DNA insert and approximately 1Kb fragments of the 5’ and 3’ flanking border regions within TE1 and TE2 were sequenced. Furthermore, parental loci within the isogenic non-transgenic lines representing the genetic background of TE1 and TE2 were sequenced to identify whether any sequence re-arrangements took place at insertion site during transformation. Entire T-DNA of the single event constituents of the breeding stack, TE1 x TE2 were also re-sequenced. The purpose was to identify whether breeding process incurs any potential changes to T-DNAs of TE1 and TE2 single events when they brought together into one background. T-DNA inserts of single events in TE1 x TE2 stack and the parental locus for each trait were PCR-amplified in overlapping fragments. The fragments were cloned and sequenced by traditional Sanger sequencing. Sequencing was followed by assembly and generation of a consensus sequence spanning the entire locus and flanking border sequences. The resulting consensus sequence was aligned to previously determined sequence of the transformation plasmid for each trait.

### Library preparation for Whole Genome Sequencing

Whole Genome Sequencing Library was prepared using TruSeq library kit (Illumina, San Diego, CA). Genomic DNA was fragmented to 800 bp by using Covaris E220 Focused-ultrasonicator (Covaris, Inc., Woburn, MA) with 105 Watt Peak Incident Power, 5% Duty Factor, 200 Cycles per burst, 50 seconds Treatment Time. The sheared double-stranded DNA was end-repaired, A- tailed, ligated to trueSeq index adapters according to manufacturer’s protocol (TruSeq DNA Sample Preparation Guide). Size selection was performed for 800 bp by Gel size selection method and size selected DNA fragments were enriched by PCR. The final library is validated using Bioanalyzer 2100 High Sensitivity DNA chip (Agilent Technologies, Santa Clara, CA) and Qubit 2.0 Fluorometer High Sensitivity kit (Life technologies, Austin, TX). The libraries were pooled and diluted to 2nM. PE sequencing was done using Illumina’s HiSeq2000 (Illumina, San Diego, CA) instrument following the manufacturer’s protocol that produced paired-end short sequence reads (approximately 100 bp long).

### Library preparation for targeted capture sequencing

Final Illumina libraries used for whole genome sequencing were pooled together equally for a total of 1.2 μg for one targeted capture reaction. The libraries attached to the custom probes were pulled down by capture beads. Unbound fragments were removed by washing and PCR was performed to enrich libraries attached to the capture beads. PCR product was validated using Bioanalyzer 2100 High sensitivity DNA chip (Agilent Technologies, city, Austin, TX) and Qubit 2.0 Fluorometer High Sensitivity kit (Life Technologies, Austin, TX).

Final library was diluted to 2nM concentration for sequencing. PE sequencing was done using Illumina’s MiSeq (Illumina, San Diego, CA) instrument following the manufacturer’s protocol that produced paired-end short sequence reads (approximately 250 bp long).

### Sequencing quality and assembly

Initial Quality control of the sequenced reads was done using the CASAVA software (Illumina, Inc. San Diego, CA). Following that, the reads were trimmed for adapter sequences, and all reads with Phred quality scores below 30 (Q30) were discarded. The trimmed reads were mapped to the Soybean Williams82 reference genome sequence using software packages, including Burrows-Wheeler Aligner [23] and Samtools [24]. Sequence coverage for genome and transgene was obtained using BEDTools [25]. Custom scripts were used to extract the junction reads. The figures presented here were made using Circos [26].

## Supporting Information

S1 Fig Map of transformation plasmid containing T-DNA with three expression cassettes for Transgenic Event 1 (TE1). The transgenic insert contains ten regulatory elements, designated as T1_En, representing three expression cassettes, where T1_E1 is an expression enhancer; T1_E4, T1_E5, and T1_E8 are promoters; T1_E3, T1_E6, and T1_E9 are genes of interest (GOI); T1_E2, T1_E7 and T1_E10 are terminators. Blue bars represent the probes designed to carry our Southern Blot analysis. T1 probes 1, 2, and 3 are GOI-based probes, whereas BB1, 2, and 3 are backbone probes.
(TIF)

S2 Fig Map of transformation plasmid containing T-DNA with three expression cassettes for Transgenic event 2 (TE2). The transgenic insert contains nine regulatory elements, designated as T2_En, representing three expression cassettes, where T2_E1, T2_E4, and T2_E7 are promoters; T2_E2, T1_E5, and T2_E8 are genes of interest (GOI); T2_E3, T2_E6 and T1_E9 are terminators. Blue bars represent the probes designed to carry our Southern Blot analysis. T2 probes 1, 2, and 3 are GOI-based probes, whereas T2 BB1, 2, and 3 are backbone probes.
(TIF)

S3 Fig Southern blot analysis of TE1. Lanes 1 and 20 are molecular weight markers. Lane 2 is non-transgenic control spiked with plasmid DNA of TE1. Lane 3 is non-transgenic control. Lane 4–19 are transgenic TE1 single events from three generations with three replications for each generation. (A) Blot image with *Msc*I digestion and T1 Probe 1 hybridization (B) Blot image with *Not*I/*Sph*I digestion and T1 Probe 2 hybridization (C) Blot image with *Msc*I digestion and T1 Probe 3 hybridization.
(TIF)

S4 Fig Southern blot analysis of TE2. Lane 1 and Lane 20 are molecular weight markers. Lane 2 is non-transgenic control spiked with ptrait2. Lane 3 is non-transgenic control. Lane 4–19 are transgenic TE2 single events from three generations with three replications for each generation (A): T2 Probe 1 (B) T2 Probe 2 (C) T2 Probe 3.
(TIF)

S5 Fig Southern blot analysis of TE1. Lane 1 and Lane 20 are molecular weight markers. Lane 2 is non-transgenic control spiked with ptrait1. Lane 3 is non-transgenic control. Lane 4–19 are transgenic TE1 single events from three generations with three replications for each generation. Backbone probes: (A) BB1 Probe (B) BB2 Probe (C) BB3 Probe.
(TIF)

S6 Fig Southern blot analysis of TE2. Lane 1 and Lane 20 are molecular weight markers. Lane 2 is non-transgenic control spiked with pTE2. Lane 3 is non-transgenic control. Lane 4–19 are transgenic TE2 single events from three generations with three replications for each generation Backbone probes: (A) BB1 Probe (B) BB2 Probe (C) BB3 Probe.
(TIF)

S7 Fig Southern blot analysis of stacked event (TE1 x TE2). Lane 1 and Lane 10 are molecular weight markers. Lane 2 is non-transgenic control spiked with pTE1. Lane 3 is non-transgenic control spiked with pTE2. Lane 4 is non-transgenic control. Lane 5 is transgenic single event (TE1), Lane 6 is transgenic single event (TE 2), Lane 7–9 are stacked event (TE1 x TE2) from homozygous line with three replications. (A) TE1 Probe 1 (B) TE2 Probe 2 (C) TE1 Probe 3 and TE2 Probe 3.
(TIF)

S8 Fig Sanger sequencing of Single event TE1, TE2, and Stack (TE1 x TE2). **A.** Comparison of T-DNA region of plasmid of TE 1 and T-DNA inserted in TE 1. A 3 bp insertion in T-DNA of TE 1 is shown in red. Dotted blue indicates intended T-DNA from plasmid to actual T-DNA inserted in TE 1. B. Comparison of T-DNA region of plasmid of TE 2 and T-DNA inserted in TE 2. A 135 bp insertion in 5’ and 9 bp insertion at 3’ of T-DNA inserted in TE 2 are shown in red. C. Comparison of Single event TE 1 and TE 1 in Stack. Dotted blue indicates actual T-DNA inserted single and stack. D. Comparison of Single event TE 2 and TE 2 in Stack. Dotted blue indicates actual T-DNA inserted single and stack.
(TIF)
