# Supplementary figures and images for: Molecular Characterization of Transgenic Events Using Next Generation Sequencing Approach
Source: PLoS One. 2016 Feb 23;11(2):e0149515. doi: 10.1371/journal.pone.0149515 (PMC4764375; doi:10.1371/journal.pone.0149515)

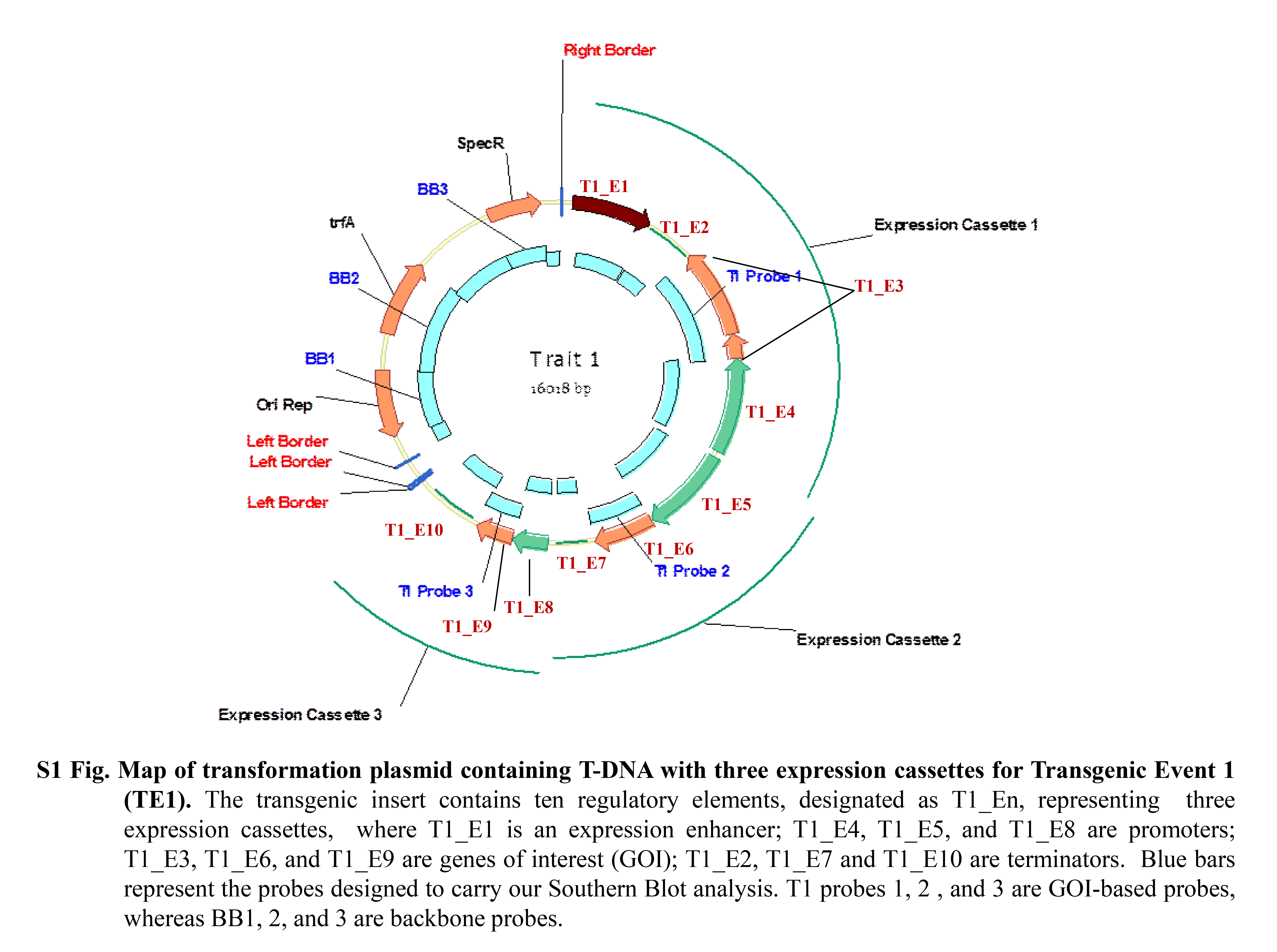

Supplement: S1 Fig — The transgenic insert contains ten regulatory elements, designated as T1_En, representing three expression cassettes, where T1_E1 is an expression enhancer; T1_E4, T1_E5, and T1_E8 are promoters; T1_E3, T1_E6, and T1_E9 are genes of interest (GOI); T1_E2, T1_E7 and T1_E10 are terminators. Blue bars represent the probes designed to carry our Southern Blot analysis. T1 probes 1, 2, and 3 are GOI-based probes, whereas BB1, 2, and 3 are backbone probes. (TIF) [file pone.0149515.s001.tif]

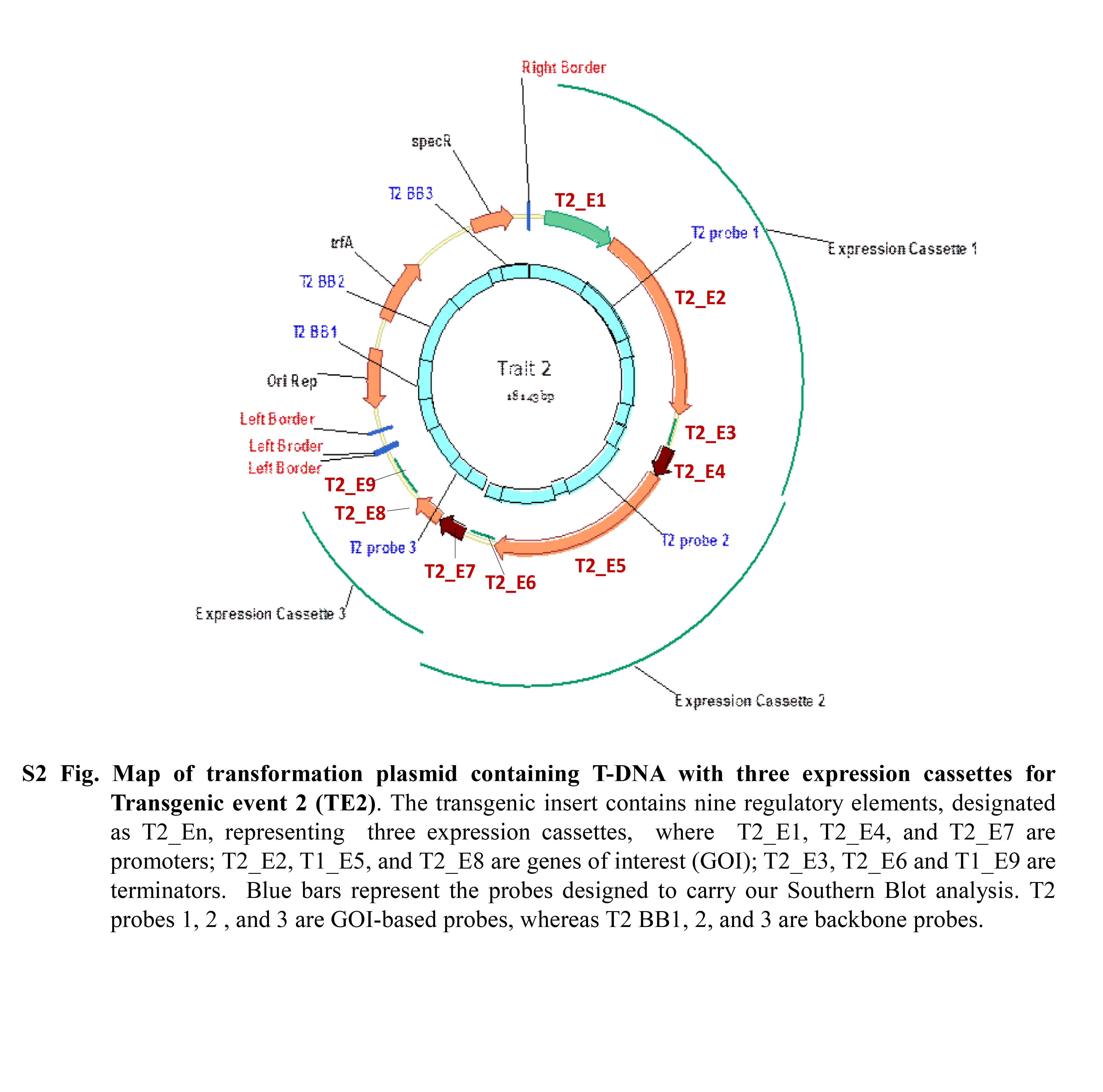

Supplement: S2 Fig — The transgenic insert contains nine regulatory elements, designated as T2_En, representing three expression cassettes, where T2_E1, T2_E4, and T2_E7 are promoters; T2_E2, T1_E5, and T2_E8 are genes of interest (GOI); T2_E3, T2_E6 and T1_E9 are terminators. Blue bars represent the probes designed to carry our Southern Blot analysis. T2 probes 1, 2, and 3 are GOI-based probes, whereas T2 BB1, 2, and 3 are backbone probes. (TIF) [file pone.0149515.s002.tif]

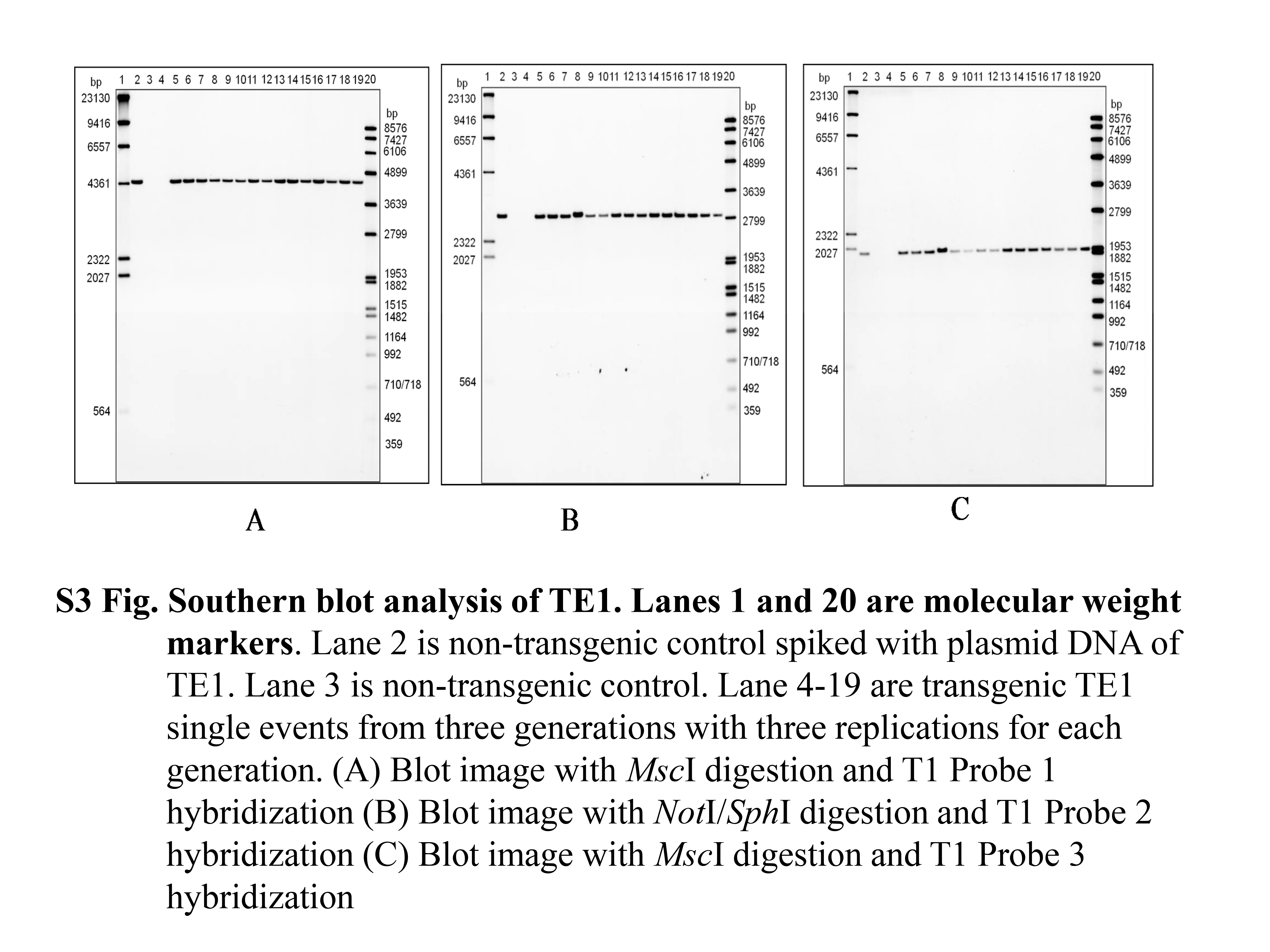

Supplement: S3 Fig — Lanes 1 and 20 are molecular weight markers. Lane 2 is non-transgenic control spiked with plasmid DNA of TE1. Lane 3 is non-transgenic control. Lane 4–19 are transgenic TE1 single events from three generations with three replications for each generation. (A) Blot image with MscI digestion and T1 Probe 1 hybridization (B) Blot image with NotI/SphI digestion and T1 Probe 2 hybridization (C) Blot image with MscI digestion and T1 Probe 3 hybridization. (TIF) [file pone.0149515.s003.tif]

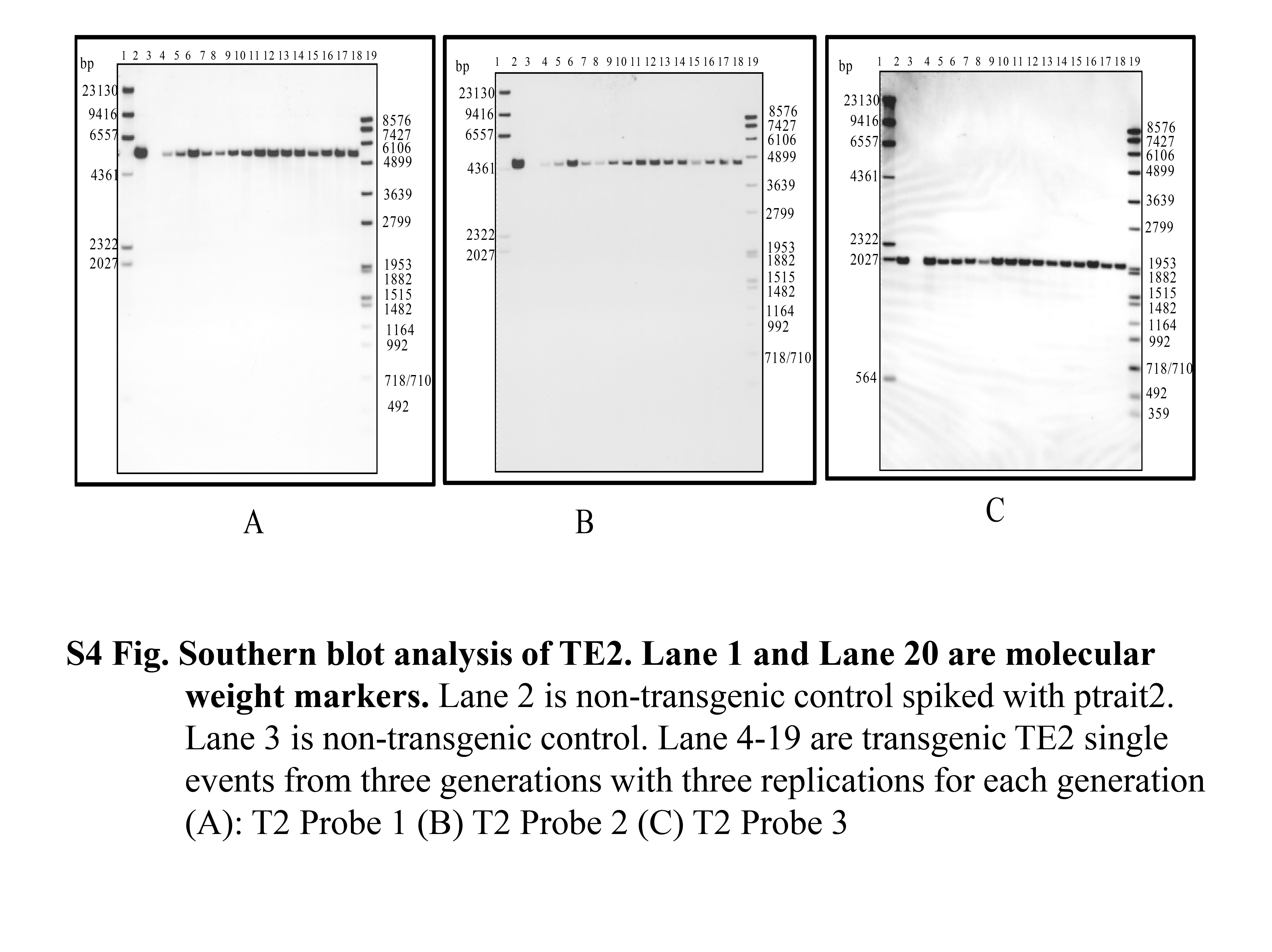

Supplement: S4 Fig — Lane 1 and Lane 20 are molecular weight markers. Lane 2 is non-transgenic control spiked with ptrait2. Lane 3 is non-transgenic control. Lane 4–19 are transgenic TE2 single events from three generations with three replications for each generation (A): T2 Probe 1 (B) T2 Probe 2 (C) T2 Probe 3. (TIF) [file pone.0149515.s004.tif]

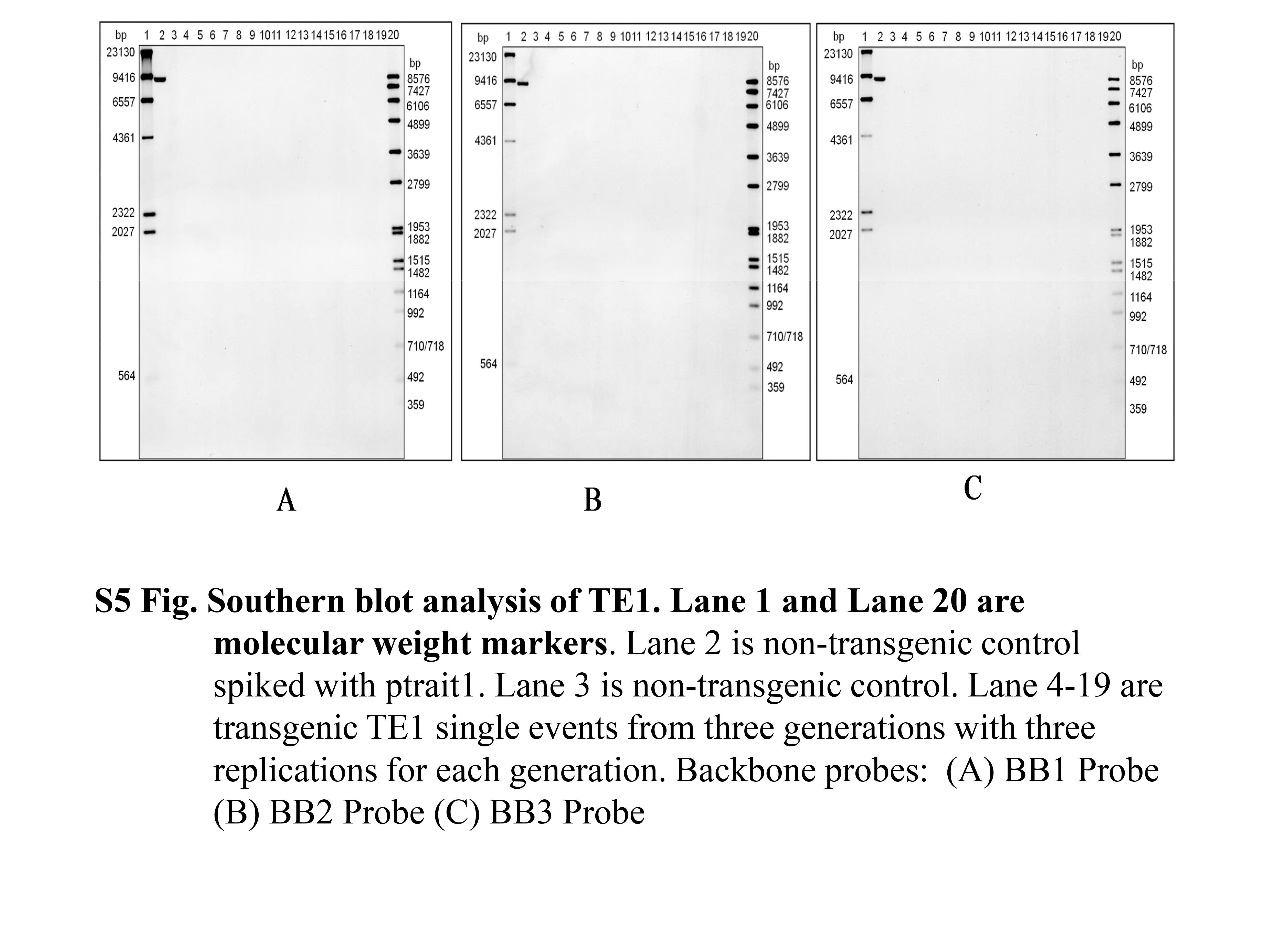

Supplement: S5 Fig — Lane 1 and Lane 20 are molecular weight markers. Lane 2 is non-transgenic control spiked with ptrait1. Lane 3 is non-transgenic control. Lane 4–19 are transgenic TE1 single events from three generations with three replications for each generation. Backbone probes: (A) BB1 Probe (B) BB2 Probe (C) BB3 Probe. (TIF) [file pone.0149515.s005.tif]

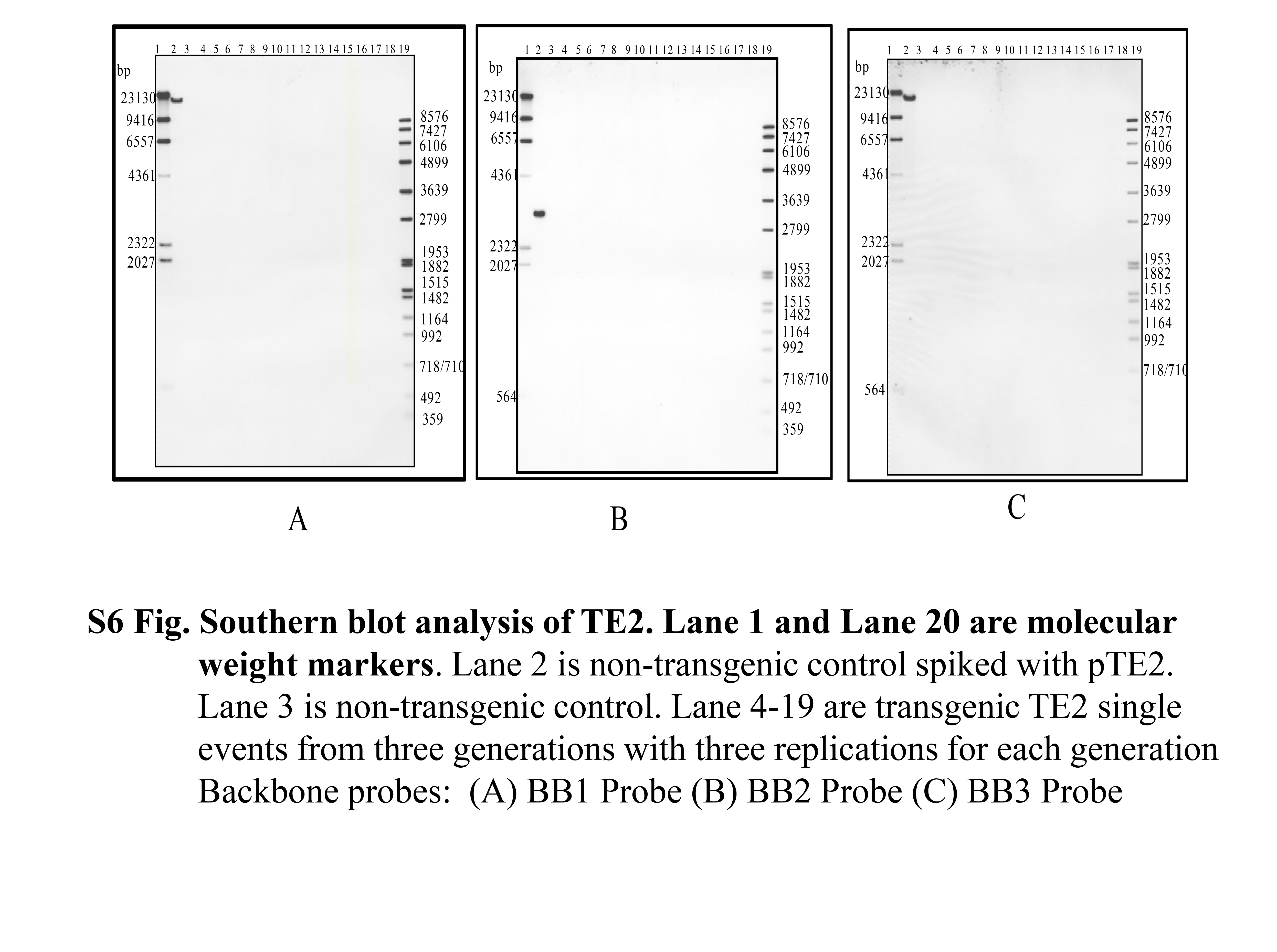

Supplement: S6 Fig — Lane 1 and Lane 20 are molecular weight markers. Lane 2 is non-transgenic control spiked with pTE2. Lane 3 is non-transgenic control. Lane 4–19 are transgenic TE2 single events from three generations with three replications for each generation Backbone probes: (A) BB1 Probe (B) BB2 Probe (C) BB3 Probe. (TIF) [file pone.0149515.s006.tif]

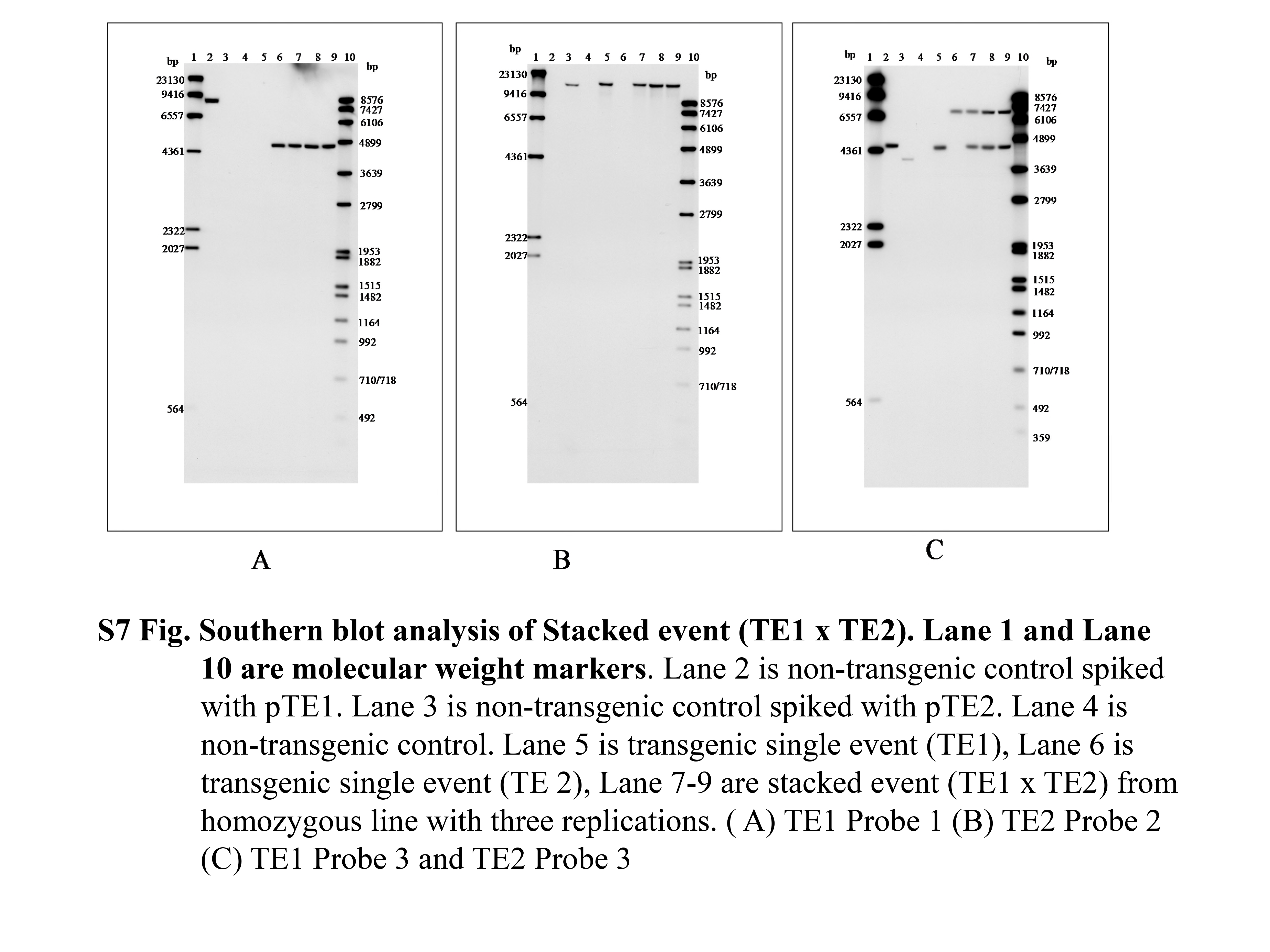

Supplement: S7 Fig — Lane 1 and Lane 10 are molecular weight markers. Lane 2 is non-transgenic control spiked with pTE1. Lane 3 is non-transgenic control spiked with pTE2. Lane 4 is non-transgenic control. Lane 5 is transgenic single event (TE1), Lane 6 is transgenic single event (TE 2), Lane 7–9 are stacked event (TE1 x TE2) from homozygous line with three replications. (A) TE1 Probe 1 (B) TE2 Probe 2 (C) TE1 Probe 3 and TE2 Probe 3. (TIF) [file pone.0149515.s007.tif]

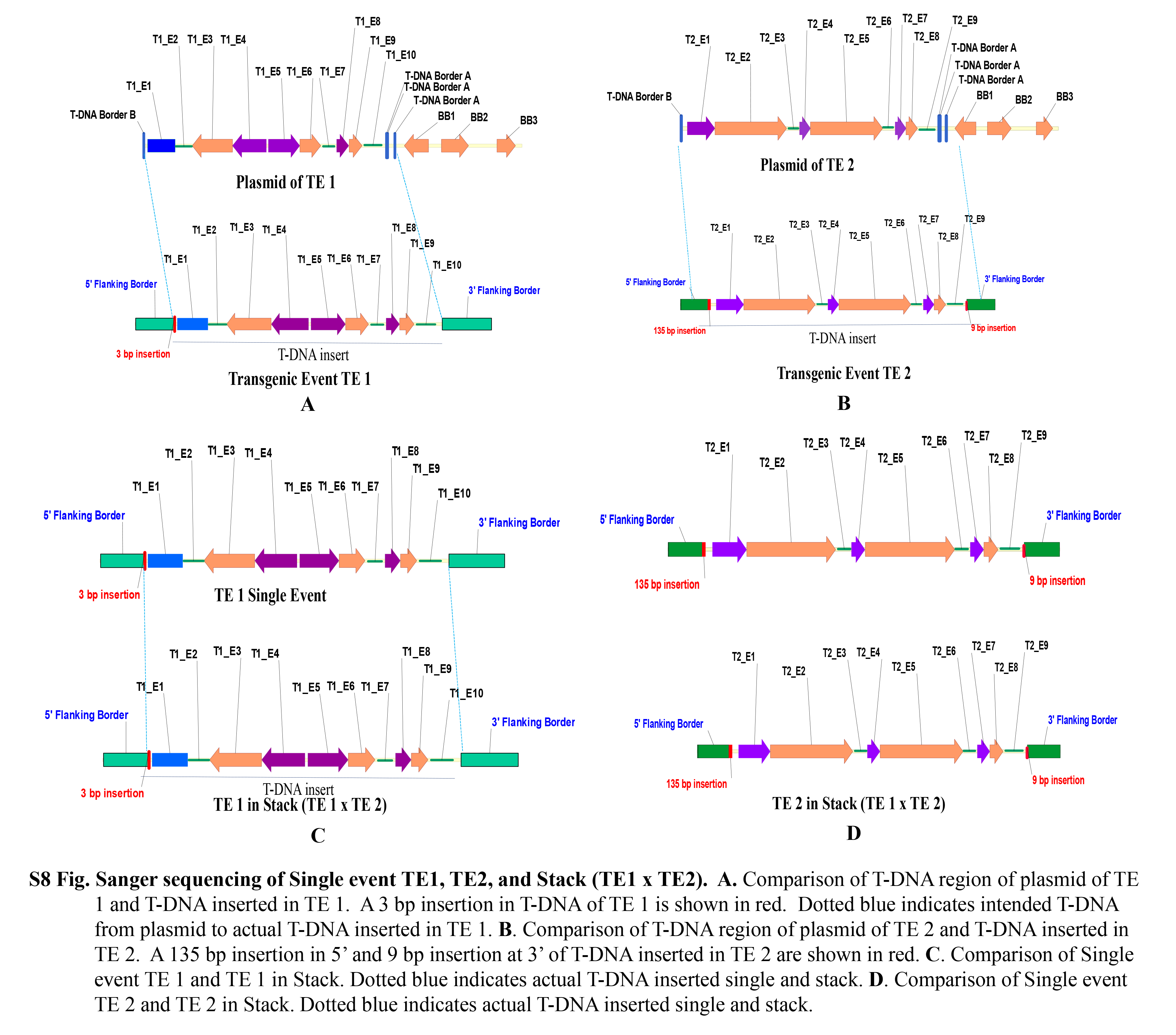

Supplement: S8 Fig — A. Comparison of T-DNA region of plasmid of TE 1 and T-DNA inserted in TE 1. A 3 bp insertion in T-DNA of TE 1 is shown in red. Dotted blue indicates intended T-DNA from plasmid to actual T-DNA inserted in TE 1. B. Comparison of T-DNA region of plasmid of TE 2 and T-DNA inserted in TE 2. A 135 bp insertion in 5’ and 9 bp insertion at 3’ of T-DNA inserted in TE 2 are shown in red. C. Comparison of Single event TE 1 and TE 1 in Stack. Dotted blue indicates actual T-DNA inserted single and stack. D. Comparison of Single event TE 2 and TE 2 in Stack. Dotted blue indicates actual T-DNA inserted single and stack. (TIF) [file pone.0149515.s008.tif]
